# Supplementary material for: Heparin-based hydrogel scaffolding alters the transcriptomic profile and increases the chemoresistance of MDA-MB-231 triple-negative breast cancer cells
Source: Biomater Sci. 2020 Feb 13;8(10):2786–96. doi: 10.1039/c9bm01481k (PMC7497406; doi:10.1039/c9bm01481k)
Supplement: Supplementary file 2 [file BM-008-C9BM01481K-s002.zip › Supplementary File 4/HP-B hydrogel + EGF (s)vControl/Pathways/my_analysis.Gsea.1545201170987/HALLMARK_ALLOGRAFT_REJECTION.html]

Details for gene set HALLMARK\_ALLOGRAFT\_REJECTION[GSEA]

|  || Dataset | expr.class.cls#HYDEGF\_versus\_CONTROL.class.cls#HYDEGF\_versus\_CONTROL\_repos |
| Phenotype | class.cls#HYDEGF\_versus\_CONTROL\_repos |
| Upregulated in class | CONTROL |
| GeneSet | HALLMARK\_ALLOGRAFT\_REJECTION |
| Enrichment Score (ES) | -0.48609507 |
| Normalized Enrichment Score (NES) | -2.1575227 |
| Nominal p-value | 0.0 |
| FDR q-value | 0.0 |
| FWER p-Value | 0.0 |
Table: GSEA Results Summary

  

Fig 1: Enrichment plot: HALLMARK\_ALLOGRAFT\_REJECTION      
 Profile of the Running ES Score & Positions of GeneSet Members on the Rank Ordered List

  

| PROBE | DESCRIPTION (from dataset) | GENE SYMBOL | GENE\_TITLE | RANK IN GENE LIST | RANK METRIC SCORE | RUNNING ES | CORE ENRICHMENT || 1 | IL11 | na |  |  | 654 | 1.946 | -0.0152 | No |
| 2 | TRAF2 | na |  |  | 932 | 1.730 | -0.0128 | No |
| 3 | EGFR | na |  |  | 1395 | 1.470 | -0.0226 | No |
| 4 | HLA-DOB | na |  |  | 1480 | 1.440 | -0.0130 | No |
| 5 | AKT1 | na |  |  | 2221 | 1.205 | -0.0399 | No |
| 6 | TLR6 | na |  |  | 2323 | 1.171 | -0.0338 | No |
| 7 | IRF7 | na |  |  | 2931 | 1.020 | -0.0556 | No |
| 8 | IKBKB | na |  |  | 3023 | 0.999 | -0.0506 | No |
| 9 | PRKCG | na |  |  | 3395 | 0.922 | -0.0610 | No |
| 10 | CD8A | na |  |  | 4082 | 0.799 | -0.0892 | No |
| 11 | AARS | na |  |  | 4113 | 0.795 | -0.0829 | No |
| 12 | JAK2 | na |  |  | 4877 | 0.671 | -0.1164 | No |
| 13 | ELF4 | na |  |  | 5121 | 0.633 | -0.1229 | No |
| 14 | LIF | na |  |  | 5147 | 0.629 | -0.1180 | No |
| 15 | IGSF6 | na |  |  | 5483 | 0.578 | -0.1299 | No |
| 16 | PTPN6 | na |  |  | 5592 | 0.564 | -0.1301 | No |
| 17 | BRCA1 | na |  |  | 6597 | 0.424 | -0.1785 | No |
| 18 | EIF3D | na |  |  | 6748 | 0.404 | -0.1825 | No |
| 19 | HCLS1 | na |  |  | 6985 | 0.373 | -0.1912 | No |
| 20 | CSK | na |  |  | 7045 | 0.366 | -0.1907 | No |
| 21 | IL7 | na |  |  | 7239 | 0.338 | -0.1975 | No |
| 22 | IL12A | na |  |  | 7348 | 0.322 | -0.2000 | No |
| 23 | IL18 | na |  |  | 7482 | 0.306 | -0.2040 | No |
| 24 | RPS9 | na |  |  | 7541 | 0.300 | -0.2041 | No |
| 25 | IL27RA | na |  |  | 7656 | 0.281 | -0.2073 | No |
| 26 | NLRP3 | na |  |  | 7930 | 0.252 | -0.2191 | No |
| 27 | IFNAR2 | na |  |  | 8233 | 0.217 | -0.2328 | No |
| 28 | HDAC9 | na |  |  | 8291 | 0.210 | -0.2338 | No |
| 29 | DYRK3 | na |  |  | 8449 | 0.195 | -0.2401 | No |
| 30 | ITGB2 | na |  |  | 8519 | 0.184 | -0.2419 | No |
| 31 | INHBB | na |  |  | 8618 | 0.169 | -0.2454 | No |
| 32 | RPS19 | na |  |  | 8810 | 0.146 | -0.2539 | No |
| 33 | CCND3 | na |  |  | 8864 | 0.140 | -0.2554 | No |
| 34 | EIF4G3 | na |  |  | 9150 | 0.109 | -0.2692 | No |
| 35 | EIF3A | na |  |  | 9362 | 0.080 | -0.2795 | No |
| 36 | GCNT1 | na |  |  | 10315 | -0.025 | -0.3291 | No |
| 37 | TLR2 | na |  |  | 10870 | -0.089 | -0.3573 | No |
| 38 | MTIF2 | na |  |  | 10929 | -0.099 | -0.3594 | No |
| 39 | RIPK2 | na |  |  | 11399 | -0.146 | -0.3825 | No |
| 40 | STAT1 | na |  |  | 11412 | -0.148 | -0.3817 | No |
| 41 | NME1 | na |  |  | 12003 | -0.217 | -0.4105 | No |
| 42 | ABCE1 | na |  |  | 12033 | -0.222 | -0.4098 | No |
| 43 | IL4R | na |  |  | 12312 | -0.263 | -0.4218 | No |
| 44 | BCAT1 | na |  |  | 12337 | -0.267 | -0.4205 | No |
| 45 | RARS | na |  |  | 12359 | -0.269 | -0.4190 | No |
| 46 | GLMN | na |  |  | 12653 | -0.306 | -0.4313 | No |
| 47 | IL15 | na |  |  | 12671 | -0.309 | -0.4292 | No |
| 48 | LY75 | na |  |  | 12819 | -0.332 | -0.4336 | No |
| 49 | TLR3 | na |  |  | 12974 | -0.336 | -0.4384 | No |
| 50 | CAPG | na |  |  | 12979 | -0.337 | -0.4353 | No |
| 51 | TAP2 | na |  |  | 13058 | -0.351 | -0.4360 | No |
| 52 | PSMB10 | na |  |  | 13119 | -0.356 | -0.4357 | No |
| 53 | CD40 | na |  |  | 13369 | -0.398 | -0.4448 | No |
| 54 | ACVR2A | na |  |  | 13516 | -0.416 | -0.4484 | No |
| 55 | TLR1 | na |  |  | 13598 | -0.425 | -0.4485 | No |
| 56 | FAS | na |  |  | 13787 | -0.456 | -0.4539 | No |
| 57 | ACHE | na |  |  | 13812 | -0.461 | -0.4506 | No |
| 58 | TGFB1 | na |  |  | 14260 | -0.515 | -0.4690 | No |
| 59 | APBB1 | na |  |  | 14337 | -0.526 | -0.4679 | No |
| 60 | CCL2 | na |  |  | 14503 | -0.557 | -0.4710 | No |
| 61 | CCND2 | na |  |  | 14645 | -0.580 | -0.4728 | No |
| 62 | INHBA | na |  |  | 14670 | -0.585 | -0.4683 | No |
| 63 | HLA-DQA1 | na |  |  | 14720 | -0.591 | -0.4651 | No |
| 64 | TNF | na |  |  | 14846 | -0.611 | -0.4656 | No |
| 65 | SOCS5 | na |  |  | 14904 | -0.619 | -0.4626 | No |
| 66 | ETS1 | na |  |  | 15068 | -0.646 | -0.4648 | No |
| 67 | DARS | na |  |  | 15201 | -0.669 | -0.4652 | No |
| 68 | GALNT1 | na |  |  | 15251 | -0.679 | -0.4611 | No |
| 69 | ABI1 | na |  |  | 15459 | -0.714 | -0.4649 | No |
| 70 | EIF5A | na |  |  | 15490 | -0.720 | -0.4595 | No |
| 71 | MRPL3 | na |  |  | 15999 | -0.818 | -0.4781 | Yes |
| 72 | EIF3J | na |  |  | 16142 | -0.855 | -0.4772 | Yes |
| 73 | MAP3K7 | na |  |  | 16187 | -0.867 | -0.4710 | Yes |
| 74 | TPD52 | na |  |  | 16281 | -0.893 | -0.4671 | Yes |
| 75 | TAPBP | na |  |  | 16305 | -0.899 | -0.4595 | Yes |
| 76 | LYN | na |  |  | 16413 | -0.926 | -0.4561 | Yes |
| 77 | ICOSLG | na |  |  | 16433 | -0.928 | -0.4480 | Yes |
| 78 | TGFB2 | na |  |  | 16607 | -0.972 | -0.4475 | Yes |
| 79 | HLA-DMB | na |  |  | 16649 | -0.985 | -0.4401 | Yes |
| 80 | TIMP1 | na |  |  | 16662 | -0.990 | -0.4310 | Yes |
| 81 | RPL9 | na |  |  | 16702 | -1.000 | -0.4233 | Yes |
| 82 | UBE2N | na |  |  | 16783 | -1.022 | -0.4174 | Yes |
| 83 | F2R | na |  |  | 16819 | -1.032 | -0.4092 | Yes |
| 84 | TAP1 | na |  |  | 16995 | -1.089 | -0.4077 | Yes |
| 85 | HLA-G | na |  |  | 17031 | -1.100 | -0.3988 | Yes |
| 86 | ICAM1 | na |  |  | 17147 | -1.127 | -0.3938 | Yes |
| 87 | SRGN | na |  |  | 17167 | -1.133 | -0.3837 | Yes |
| 88 | HLA-E | na |  |  | 17182 | -1.138 | -0.3733 | Yes |
| 89 | HLA-DMA | na |  |  | 17221 | -1.152 | -0.3640 | Yes |
| 90 | CFP | na |  |  | 17242 | -1.156 | -0.3537 | Yes |
| 91 | HIF1A | na |  |  | 17317 | -1.190 | -0.3459 | Yes |
| 92 | BCL3 | na |  |  | 17353 | -1.200 | -0.3360 | Yes |
| 93 | C2 | na |  |  | 17358 | -1.202 | -0.3245 | Yes |
| 94 | NCK1 | na |  |  | 17360 | -1.202 | -0.3128 | Yes |
| 95 | WARS | na |  |  | 17398 | -1.213 | -0.3029 | Yes |
| 96 | CRTAM | na |  |  | 17429 | -1.227 | -0.2924 | Yes |
| 97 | RPS3A | na |  |  | 17475 | -1.248 | -0.2826 | Yes |
| 98 | SOCS1 | na |  |  | 17552 | -1.279 | -0.2740 | Yes |
| 99 | UBE2D1 | na |  |  | 17727 | -1.349 | -0.2700 | Yes |
| 100 | BCL10 | na |  |  | 17922 | -1.452 | -0.2659 | Yes |
| 101 | CD74 | na |  |  | 17961 | -1.469 | -0.2535 | Yes |
| 102 | RPL39 | na |  |  | 18037 | -1.506 | -0.2427 | Yes |
| 103 | IL1B | na |  |  | 18115 | -1.556 | -0.2315 | Yes |
| 104 | IL6 | na |  |  | 18227 | -1.619 | -0.2215 | Yes |
| 105 | IFNGR2 | na |  |  | 18361 | -1.722 | -0.2116 | Yes |
| 106 | CTSS | na |  |  | 18454 | -1.799 | -0.1988 | Yes |
| 107 | DEGS1 | na |  |  | 18462 | -1.808 | -0.1815 | Yes |
| 108 | EREG | na |  |  | 18655 | -1.994 | -0.1721 | Yes |
| 109 | HLA-DOA | na |  |  | 18767 | -2.142 | -0.1569 | Yes |
| 110 | LTB | na |  |  | 18802 | -2.196 | -0.1372 | Yes |
| 111 | HLA-DRA | na |  |  | 18824 | -2.236 | -0.1164 | Yes |
| 112 | IFNGR1 | na |  |  | 18912 | -2.376 | -0.0977 | Yes |
| 113 | ST8SIA4 | na |  |  | 19018 | -2.638 | -0.0774 | Yes |
| 114 | GBP2 | na |  |  | 19029 | -2.666 | -0.0519 | Yes |
| 115 | CD47 | na |  |  | 19123 | -3.029 | -0.0271 | Yes |
| 116 | B2M | na |  |  | 19139 | -3.164 | 0.0031 | Yes |
Table: GSEA details [plain text format]

  

Fig 2: HALLMARK\_ALLOGRAFT\_REJECTION      
 Blue-Pink O' Gram in the Space of the Analyzed GeneSet

  

Fig 3: HALLMARK\_ALLOGRAFT\_REJECTION: Random ES distribution      
 Gene set null distribution of ES for **HALLMARK\_ALLOGRAFT\_REJECTION**
